# Randomized multicenter trial on the effect of radiotherapy for plantar Fasciitis (painful heel spur) using very low doses – a study protocol

**DOI:** 10.1186/1748-717X-3-27

**Published:** 2008-09-18

**Authors:** Marcus Niewald, M Heinrich Seegenschmiedt, Oliver Micke, Stefan Gräber, the GCGBD (German cooperative group on the radiotherapy for benign diseases) of the DEGRO (German society for radiation oncology)

**Affiliations:** 1 Department of Radiooncology, Saarland University Hospital, Kirrberger Str. 1, D-66421 Homburg, Germany; 2 Deptartment of Radiooncology, Alfried Krupp Hospital, Alfried-Krupp-Str. 21, D-45117 Essen, Germany; 3 Department of Radiooncology, St. Franziskus Hospital, Kiskerstr. 26, D-33615 Bielefeld, Germany; 4 Institute for Medical Biometrics, Epidemiology and Medical Informatics, Saarland University Hospital, Kirrberger Str. 1, D-66421 Homburg, Germany

## Abstract

**Background:**

A lot of retrospective data concerning the effect of radiotherapy on the painful heel spur (plantar fasciitis) is available in the literature. Nevertheless, a randomized proof of this effect is still missing. Thus, the GCGBD (German cooperative group on radiotherapy for benign diseases) of the DEGRO (German Society for Radiation Oncology) decided to start a randomized multicenter trial in order to find out if the effect of a conventional total dose is superior compared to that of a very low dose.

**Methods/Design:**

In a prospective, controlled and randomized phase III trial two radiotherapy schedules are to be compared:

standard arm: total dose 6.0 Gy in single fractions of 1.0 Gy applied twice a week

experimental arm: total dose 0.6 Gy in single fractions of 0.1 Gy applied twice a week (acting as a placebo)

Patients aged over 40 years who have been diagnosed clinically and radiologically to be suffering from a painful heel spur for at least six months can be included. Former trauma, surgery or radiotherapy to the heel are not allowed nor are patients with a severe psychiatric disease or women during pregnancy and breastfeeding. According to the statistical power calculation 100 patients have to be enrolled into each arm.

After having obtaining a written informed consent a patient is randomized by the statistician to one of the arms mentioned above. After radiotherapy, the patients are seen first every six weeks, then regularly up to 48 months after therapy, they additionally receive a questionnaire every six weeks after the follow-up examinations.

The effect is measured using several target variables (scores): Calcaneodynia-score according to Rowe et al., SF-12 score, and visual analogue scale of pain. The most important endpoint is the pain relief three months after therapy. Patients with an inadequate result are offered a second radiotherapy series applying the standard dose (equally in both arms).

This trial protocol has been approved by the expert panel of the DEGRO as well as by the Ethics committee of the Saarland Physicians' Chamber. The trial is supported by a HOMFOR grant (Saarland University Research Grant).

**Trial registration:**

Current controlled trials ISRCTN94220918

## Background

### Introduction

To our knowledge, the painful heel spur was first described by Plettner et al.[1] in 1900 summarizing their radiological findings of exostoses situated at the plantar part of the calcaneus or at the insertion point of the plantar aponeurosis. Various authors give values for the incidence of 8 to 88% of an unselected population [2-4]. Risk factors may be old age, obesity, and foot or leg deformities. Histopathologically, the heel spur is a fibroostosis promoted by mechanical stress to the plantar aponeurosis, slowly and continuously growing into its insertion region[5]. In a more chronical stage of the disease, the degenerative changes cause a local inflammation of the plantar aponeurosis (plantar fasciitis), which should be well differentiated from – for example – rheumatoid arthritis.

### Clinical findings

Heel spurs – even when clearly visible on X-rays – often are completely asymptomatic. 16% of these patients have local pain getting worse over weeks to months under the heel, which can further extend to the foot or the lower limb. Local pressure to the medial edge of the calcaneus may be painful[5]. In our own experience, most of the patients cannot stand or walk for a long time, the pain may be even worse during the first minutes of rest after a walk.

### Radiological findings

Conventional x-rays are the gold standard in the diagnosis of a heel spur, usually lateral pictures of the calcaneus are taken. They show a calcified spur at the inferior side of the calcaneus. The intensity of pain is not regarded to be dependent on the size of the spur.

Additionally a local ultrasound examination can be performed in order to examine the swelling and irritation of the plantar fascia. A bone scan may be positive showing local inflammation which remits after successful therapy.

### Conventional therapy methods

A great variety of therapy methods have been tested in the past but none of them has provided a high level of evidence. Ice, heat, ultrasound, radiofrequency, laser beams and extracorporal shock wave therapy have been applied. Steroids and local anesthetics injected into the plantar fascia, and oral analgetic medication (NSAID) have been prescribed. Immobilization of the foot using special splints and adjustable shoes were applied. Physiotherapy was performed[2,3].

Iontophoresis using dexamethasone was found to be superior to iontophoresis with placebo (NaCl)[6]. Extracorporal shock wave therapy yielded a complete pain relief in up to 68% of the patients[7]. A randomized trial published by Batt et al.[8] showed that better results were recorded by adding local immobilization of the foot in maximum dorsal flexion during night to a standard therapy (NSAID, splints) compared to this standard therapy alone. According to Powell et al.[9], a splint applied immediately after diagnosis was as effective as an application one month later. In further randomized trials mechanical therapy using ortheses was found better than application of local analgesics only, silicon shoe inserts were found superior to other shoe adjustments[10], functional foot ortheses were found to be more advantageous than simple heel splints[11]. Most of these methods and their results have been summarized in a Cochrane review[12]

### Surgery

As a general consensus, patients will only undergo surgery in the case that conservative therapy methods have not yielded sufficient pain relief. Heider et al.[13] report good to excellent results in 26 out of 28 feet after surgery. Favourable results were recorded as well using endoscopic release of the plantar fascia[14]. Nevertheless, complications like fractures of the calcaneus[15] as well as negative biomechanical consequences of plantar fascia relief have been reported[16].

### Radiotherapy – experimental data

The anti-inflammatory effect of radiation therapy has been known for a long time and has been reported in numerous publications. Nevertheless, the exact mechanism is still unclear. Some of the models discussed are: Improvement of blood perfusion in the tissue due to an influence of radiation on the endothelium, release of cytokines and enzymes, influence on the local parts of the vegetative nervous system, and modification of the pH-value in the tissue [17-20]. In animal experiments, Steffen et al. noticed anti-inflammatory effects of low-dose radiotherapy (6 Gy) on antigen-induced arthritis in rabbits [21]. Hildebrandt et al. have shown that low-dose radiation effects can be explained by an influence on molecular mechanisms and inflammation mediators [22-24].

### Radiotherapy – clinical results

Numerous retrospective trials have shown that low-dose radiotherapy for the painful heel spur has a good analgetic effect, pain relief has been noticed in 65 – 90% of the patients [17,20,25-28]. However, a certain placebo effect is still under discussion [25]. Goldie et al. examined this effect in 399 patients. They found a response in 60% of the patients whether irradiated or not; these results made the effect of radiotherapy questionable [29]. The trial, however, has been criticised because of missing clearly defined endpoints: furthermore the therapy was started in an acute stage of the diseases and the authors did not wait for spontaneous pain remissions.

In the meantime, several more modern trials have shown the analgetic effect of radiotherapy. Seegenschmiedt et al. [2] performed a randomized trial treating 141 patients (170 heels) for painful heel spur using orthovoltage, comparing three radiotherapy schedules: 1 Gy/fraction up to 12 Gy, 0.3 Gy/fraction up to 3 Gy and 0.5 Gy/fraction up to 5 Gy. The overall complete pain relief was reported in 67–72% of the patients. The best results were seen after a total dose of 5 Gy. These results were confirmed by Schäfer et al. using a telecobalt machine, they achieved a complete pain relief in 58% [25]. Heyd et al. used 6 MV photon beams of a linear accelerator, they noticed a frequency of pain relief of 69% [4]. The same author group published a prospective randomized trial recently [30] comparing the effect of a total dose of 3 Gy (single fraction 0.5 Gy twice weekly) to that of a total dose of 6 Gy (single fraction 1 Gy twice weekly). Radiotherapy was reported very efficient, however a dependency from dose could not be noticed. Mücke et al. looked for prognostic factors for pain relief in a multicenter trial [31]. They found an overall response in 60.9%. Significant favourable prognostic factors for pain relief were a patient's age over 58 years, the use of megavoltage techniques and the number of therapy series required.

### Radiotherapy – side effects and risks

Physicians of other specialities sometimes refuse to refer patients to radiotherapy because of the fear of local side effects such as impairment of gonad function or induction of malignancies. But we are concerned here with low doses applied to the extremities. Neither local toxicity nor tumour induction have been reported yet [26,32,33]. The dose to the gonads is comparable to that after radiodiagnostic interventions [20,34-36].

### Radiotherapy – conclusion

Summarizing the data taken from the literature it can be concluded that a low-dose radiotherapy for painful heel spur with total doses ranging from 3–12 Gy is effective in the vast majority of patients and the side effects are negligible. However, a placebo effect cannot be excluded totally. Thus, randomised trials (like the present one) using defined criteria and scores are necessary [37].

## Design

### Inclusion criteria

- Symptoms and clinical diagnosis of a painful heel spur (tenderness of the calcaneus)

- limitation of the painless walking distance

- duration of symptoms more than six months

- radiological proof of heel spur

- facultatively ultrasound, MRT or bone scan

- Karnofsky performance index > = 70%

- Age > = 40 years

- Written informed consent

### Exclusion criteria

- previous radiotherapy to the foot

- previous trauma to the foot (fracture, rupture of tendon)

- rheumatic or vascular diseases, lymphatic edema

- pregnancy, breastfeeding

- severe psychiatric disorder

### Informed consent

Before enrolment, an informed consent is to be obtained from all patients after detailed information and explanations concerning the effect and potential toxicity of therapy, alternative therapy methods, follow-up examinations, and data protection issues.

### Therapy protocol

After enrolment and filling in the SF-12-, calcaneodynia and VAS (visual analogue scale of pain) score forms, the patient is randomly assigned to either of the following therapy protocols:

Arm A: Total dose of 6 Gy in 6 single fractions of 1 Gy applied twice weekly (standard arm)

Arm B: Total dose of 0.6 Gy in 6 single fractions of 0.1 Gy applied twice weekly (experimental arm).

The dosage in Arm B was chosen first to examine if very low doses are effective at all, second it acts as a placebo irradiation; a sham irradiation was regarded unethical.

Follow-up examinations are performed every six weeks after radiotherapy either consisting of a personal examination (6,12,24,36,48 weeks after radiotherapy) or a questionnaire (after 18,30,42 weeks): every single patient is followed-up for 48 weeks. In the case of an unfavourable response to the radiotherapy after twelve weeks or more the patients will be offered a second treatment with the same technology but applying the standard dose of 6 Gy, single fractions of 1 Gy twice weekly. Such patients remain in their arms with the result classified as unsatisfactory.

The final evaluation will be performed when 200 patients have been followed-up for 48 weeks. Interim evaluations will be performed after 100 and 150 patients.

### Primary endpoints

- SF-12 sum score [38]

- Calcaneodynia sum score [2,39]

- VAS score

### Secondary endpoints

- SF-12 single score

- Calcaneodynia single score

- Event-free interval

### Randomisation and statistics

Randomisation is performed by the statistician (S.G.) as a block randomisation. The patients are assigned randomly to one therapy arm with an equal probability for both arms. 200 patients are required in order to detect a difference of 10% in the SF-12 and calcaneodynia scores (scatter 25%) with a power of 80% and an error probability of 5%.

### Radiotherapy methods

Radiotherapy is performed using orthovoltage (200–250 kV) devices, telecobalt machines or megavoltage x-ray irradiation (maximum energy 6 MV, if only higher energies are available, a bolus with a thickness of 1 cm must be applied).

Orthovoltage therapy is applied using a plantar direct field with a strip of bolus material affixed to the heel laterally and dorsally. The dose should be normed to a special reference point (for example in 5 mm tissue depth). The dose is calculated using the tables present in every department. Megavoltage therapy is performed using isocentric parallel-opposing portals, the ICRU reference point is defined in the center of the calcaneus.

The target volume should consist of the calcaneus and the plantar aponeurosis, a 2 cm wide safety margin should be added. The gonads must be shielded as well as possible.

### Quality assurance

The quality of the data is to be controlled as follows:

- Quality assurance questionnaire signed by all participants (physician and physicist)

- Visits to the centers

- Participants will be asked to send simulation x-rays or portal imaging picture as well as therapy plans of randomly selected patients

- check of the data entered into the database

### Ethics

This trial protocol has been approved by the expert panel of the DEGRO as well as by the Ethics committee of the Saarland Physicians' Chamber. The trial is supported by a HOMFOR grant (Saarland University Research Grant).

### Present status of the trial

In the meantime 49 patients have been enrolled. Ten more centers have stated their interest to participate, three of them already have the agreement of their local ethics committee.

## Competing interests

The authors declare that they have no competing interests.

## Authors' contributions

MN was responsible for the final version of this protocol in German language and wrote this manuscript. MHS had the idea to perform this trial and promoted it, he was a co-author of the German study protocol and is responsible for quality assurance procedures. OM was responsible for the former versions of the German study protocol, especially the evaluation of the literature. SG is responsible for the statistical part of this protocol and will check the final evaluation.
